# Genetic Diversity and Marker–Trait Associations in Commercial Cultivars and Weedy *Perilla frutescens* from South Korea and Japan Based on Morphological Traits and SSR Markers

**DOI:** 10.3390/plants15081273

**Published:** 2026-04-21

**Authors:** Da Hyeon Lee, Jungeun Cho, Hyeon Park, Tae Hyeon Heo, Ju Kyong Lee

**Affiliations:** 1 Department of Applied Plant Sciences, College of Agriculture and Life Sciences, Kangwon National University, Chuncheon 24341, Republic of Korea; 2 Interdisciplinary Program in Smart Agriculture, Kangwon National University, Chuncheon 24341, Republic of Korea; 3 National Institute of Agricultural Sciences, RDA, Wanju 55365, Republic of Korea

**Keywords:** *Perilla frutescens*, domestication, cultivated and weedy forms, SSR markers, association mapping, marker-assisted selection

## Abstract

Domestication has profoundly shaped the phenotypic differentiation and genetic architecture of *Perilla*. However, analyses of the morphological difference between its cultivated and weedy forms across its varieties remains incomplete. This study analyzed morphological variation, genetic diversity, population structure, and marker–trait associations of 45 accessions representing the cultivated and weedy forms of two *Perilla* varieties (*P. frutescens* var. *frutescens* and var. *crispa*) collected from South Korea and Japan. Analyses of ten qualitative and quantitative agronomic traits revealed clear domestication-related differentiation. Cultivated var. *frutescens* showed larger and heavier seeds, whereas cultivated var. *crispa* and the weedy accessions were characterized by longer inflorescences and higher floret numbers but smaller seeds. Strong positive correlations were observed among seed-related traits, particularly between seed size and seed weight (r = 0.932), indicating coordinated selection of seed traits. Genetic diversity analysis using 70 SSR markers identified 330 alleles consistent with domestication bottlenecks in cultivated forms while higher diversity was generally retained in the weedy accessions. Population structure, UPGMA clustering, and principal coordinate analyses broadly differentiated the cultivated and weedy accessions, although partial admixture indicated shared ancestry and historical gene flow. Association mapping using Q-based GLM and Q + K MLM models identified 23 significant marker–trait associations involving 16 SSR markers consistently detected across both models. Several markers were associated with multiple traits, implying pleiotropy or tight genetic linkage. Notably, five SSR markers (KNUPF192, KNUPF202, KNUPF207, KNUPF230, and KNUPF238) may represent potential candidate loci for marker-assisted selection to improve seed-related traits in var. *frutescens* and leaf-related traits in var. *crispa*.

## 1. Introduction

*Perilla frutescens* (L.) Britt. is a tetraploid species (2n = 40 = 4x) belonging to the family Lamiaceae and is predominantly self-pollinating. Native to East Asia, *Perilla* has been cultivated for centuries as both an oilseed and leafy vegetable crop [1,2,3]. Based on its morphological differentiation and patterns of utilization, *P. frutescens* is broadly classified into two major varieties: *P. frutescens* var. *frutescens* (PFF) and *P. frutescens* var. *crispa* (PFC). PFF is primarily cultivated for seed oil production, but it is also widely consumed as a leafy vegetable, particularly in South Korea. Its seeds contain approximately 46–48% oil, of which α-linolenic acid accounts for 65–75% of the total fatty acid content, making PFF one of the richest known plant sources of omega-3 fatty acids [4]. Beyond its nutritional value, recent studies have reported anticancer and immunoregulatory activities in PFF-derived compounds, highlighting its potential as a functional food crop [5]. In contrast, PFC is mainly used as a leafy vegetable and medicinal herb, especially in Japan, and is characterized by high concentrations of bioactive compounds such as rosmarinic acid, perillaldehyde, luteolin, and anthocyanins, which confer antioxidant, anti-inflammatory, and immunomodulatory properties [6]. Increasing global demand for health-promoting foods has expanded the cultivation and utilization of *Perilla* beyond East Asia, thereby enhancing its economic value and breeding importance worldwide [7,8].

Despite sharing the same chromosome number and having full inter-fertility, PFF and PFC exhibit pronounced phenotypic divergence driven by domestication and artificial selection. Key domestication-related traits, including seed size, seed coat hardness, dormancy, leaf pigmentation, and aromatic compound composition, differ between the two varieties and between their cultivated and weedy forms [9,10,11,12]. These phenotypic differences reflect underlying shifts in their genetic architecture associated with resource allocation, reproductive strategy, and secondary metabolite biosynthesis. Cultivated PFF has experienced strong selection for seed-related traits, resulting in large, soft seeds with uniform germination that facilitate mechanical harvesting and enhance oil yield and quality. In South Korea, PFF cultivars such as ‘sodam’ and ‘deulchan’ were developed for high α-linolenic acid content and are widely used for oil production, whereas ‘saebom’ was bred to maximize leaf yield for culinary purposes [13,14,15]. In contrast, cultivated PFC, although domesticated, retains many morphological features of its weedy counterpart (weedy *P. frutescens* var. *crispa*; WPFC), including small seeds with hard seed coats and a similar plant architecture [16]. As a result, cultivated PFC and WPFC are often difficult to distinguish based solely on morphological traits [16,17]. Nevertheless, PFC cultivars have been selectively bred for leaf quality and anthocyanin accumulation, leading to the development of Japanese cultivars such as ‘akarimen shiso’ and ‘chirimen red shiso’. In South Korea, intervarietal crosses between PFF and PFC have also produced anthocyanin-rich cultivars, including ‘neulbora’ and ‘saebora’ [18,19]. Weedy forms of *Perilla*, which are endemic to South Korea, represent important reservoirs of ancestral genetic variation. While weedy *P. frutescens* var. *frutescens* (WPFF) can be readily distinguished from cultivated PFF based on seed traits, cultivated PFC, particularly Japanese commercial cultivars, shows strong morphological similarity to South Korean WPFC populations. This close resemblance raises unresolved questions regarding genetic differentiation, introgression, and shared ancestry between the cultivated and weedy forms that cannot be resolved through phenotypic evaluation alone.

In minor crops such as *Perilla*, where high-quality reference genomes remain limited, simple sequence repeat (SSR) markers provide an effective framework for investigating genetic diversity, population structure, and domestication-related variation. Owing to their high polymorphism, co-dominant inheritance, and reproducibility, SSR markers remain particularly valuable for association mapping in genetically structured populations [20,21,22]. Although previous studies have developed SSR markers and examined genetic diversity in *Perilla* [23,24,25,26], most have focused on either the cultivated or weedy accessions, or on a single variety, limiting insights into shared and variety-specific genetic architectures. Moreover, association mapping linking SSR loci to domestication-related traits across both varieties and their cultivated and weedy forms remains limited [10,17,27].

This study presents the first integrative genetic analysis encompassing all four *Perilla* groups, the cultivated and weedy forms of both PFF and PFC, including commercial PFF and PFC cultivars, using a combined framework of morphological trait analysis, SSR-based genetic diversity assessment, population structure inference, and association mapping. A total of 45 *Perilla* accessions collected from South Korea and Japan were analyzed to capture the genetic variation across domesticated and weedy forms. Specifically, this study aimed to (i) identify the genetic differentiation underlying domestication-related morphological traits, (ii) resolve the population genetic structure and diversity across the cultivated and weedy forms, and (iii) detect the SSR markers associated with key agronomic traits reflecting divergent domestication strategies. By uncovering novel marker–trait associations and shared or variety-specific genetic patterns, this study advances our understanding of *Perilla* domestication and provides foundational resources for marker-assisted breeding and evolutionary research.

## 2. Results

### 2.1. Morphological Variation and Correlations Among Agronomic Traits in Cultivated and Weedy Perilla Accessions

Summary statistics for five qualitative and five quantitative traits measured in 45 cultivated and weedy *Perilla* accessions representing two *Perilla* varieties from South Korea and Japan are presented in Table 1. Comparative analysis among the four groups (PFF, PFC, WPFF, and WPFC) revealed clear morphological differentiation between the cultivated and weedy accessions.

Qualitative traits exhibited distinct patterns of variation between the cultivated and weedy forms. Adaxial (QL1) and abaxial (QL2) leaf colors varied widely among the cultivated accessions, whereas the weedy accessions showed markedly reduced variation with largely conserved pigmentation patterns. This contrast was most pronounced in WPFC, in which all of the accessions consistently displayed a purple abaxial leaf color. Flower color (QL3) was predominantly white or light pink in the PFF and WPFF accessions, while pink flowers were more frequent in the PFC and WPFC accessions. Seed color (QL4) also differed substantially between the cultivated and weedy accessions, with predominantly darker seed colors in the weedy types. Seed hardness (QL5) further distinguished the cultivated and weedy forms. The PFF cultivars exhibited both soft and hard seeds, whereas all of the WPFF, PFC, and WPFC accessions consistently displayed hard seeds. Quantitative traits also differed among four groups. Mean plant height (QN1) was 140.0 cm in PFF, 129.5 cm in PFC, 141.3 cm in WPFF, and 117.1 cm in WPFC. Inflorescence length (QN2) and number of florets (QN3) were generally higher in the weedy accessions, particularly in WPFC. In contrast, seed-related traits showed pronounced differentiation between the cultivated and weedy forms, with mean seed size (QN4) and 100-seed weight (QN5) highest in the PFF cultivars and lowest in the WPFC accessions.

**Figure 1:**
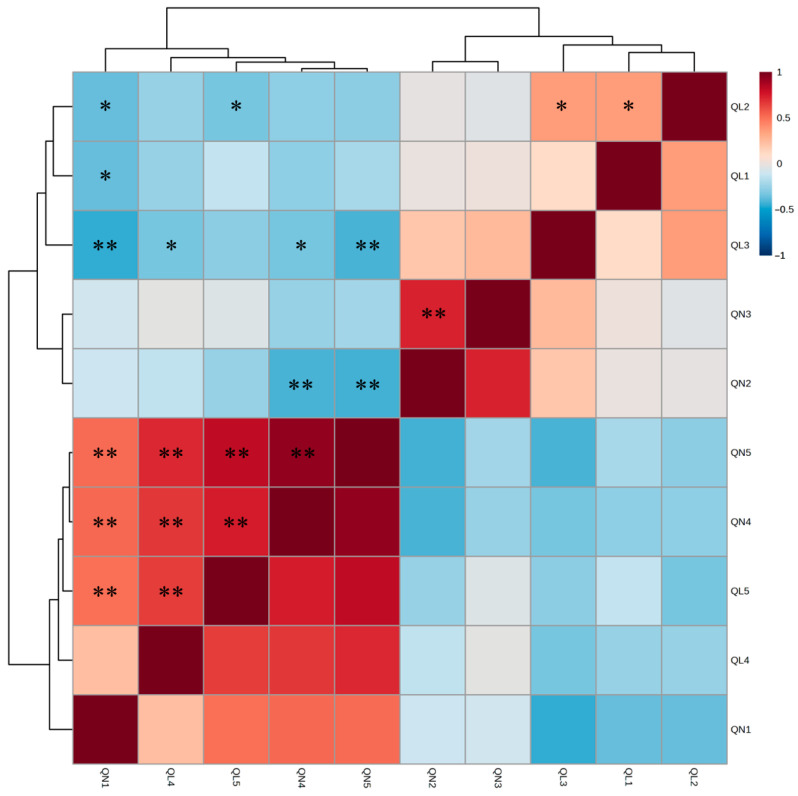
Heatmap of correlation analysis results for 10 agricultural traits in 45 *Perilla* accessions. ** Significance at *p* < 0.01. * Significance at *p* < 0.05.

Correlation analysis among the five qualitative and five quantitative traits revealed numerous significant relationships (Figure 1; Supplementary Table S1). The strongest positive correlations were observed between seed color (QL4) and seed hardness (QL5), as well as between seed size (QN4) and 100-seed weight (QN5). Several negative correlations were also detected, indicating complex interrelationships among agronomic traits.

### 2.2. Genetic Diversity and Polymorphism of SSR Loci Among 45 Perilla Accessions

A set of 70 polymorphic *Perilla* SSR loci was utilized to assess genetic diversity (GD) among the 45 accessions representing the cultivated and weedy forms of the two *Perilla* varieties. Across all of the accessions, a total of 330 alleles were detected using the 70 *Perilla* SSR markers (Table 2). The number of alleles per locus ranged from 2 to 12, with an average of 4.7 alleles per locus. The GD across the loci ranged from 0.085 to 0.845, with a mean of 0.566. Similarly, the polymorphism information content (PIC) values ranged from 0.081 to 0.829, with an average of 0.506. The major allele frequency (MAF) values varied from 0.244 to 0.956, with a mean of 0.543. Allelic frequency distribution analysis revealed that, among the 330 alleles detected, private alleles accounted for 25%, rare alleles for 34%, intermediate-frequency alleles for 27%, and abundant alleles for 13% of the total alleles (Supplementary Figure S1), indicating substantial allelic heterogeneity across the cultivated and weedy accessions.

Genetic diversity was further examined separately for the four *Perilla* groups: cultivated *P. frutescens* var. *frutescens* (PFF, n = 11), weedy var. *frutescens* (WPFF, n = 10), cultivated *P. frutescens* var. *crispa* (PFC, n = 14), and weedy var. *crispa* (WPFC, n = 10) (Supplementary Table S2). The mean number of alleles per locus was highest in WPFF (3.1), followed by WPFC (2.7), and was lower in the cultivated groups (2.4 in both PFF and PFC). Correspondingly, the mean MAF values were highest in PFF (0.726) and PFC (0.704) and lower in WPFF (0.623) and WPFC (0.681). The mean GD values were 0.375 (PFF), 0.489 (WPFF), 0.383 (PFC), and 0.406 (WPFC), while the mean PIC values were 0.322, 0.437, 0.323, and 0.356 for PFF, WPFF, PFC, and WPFC, respectively (Supplementary Table S2). Overall, the weedy accessions consistently exhibited higher genetic diversity than the cultivated accessions, consistent with domestication-associated genetic bottlenecks.

### 2.3. Population Structure Analysis and Phylogenetic Relationships Between Cultivated and Weedy Perilla Accessions from South Korea and Japan

**Figure 2:**
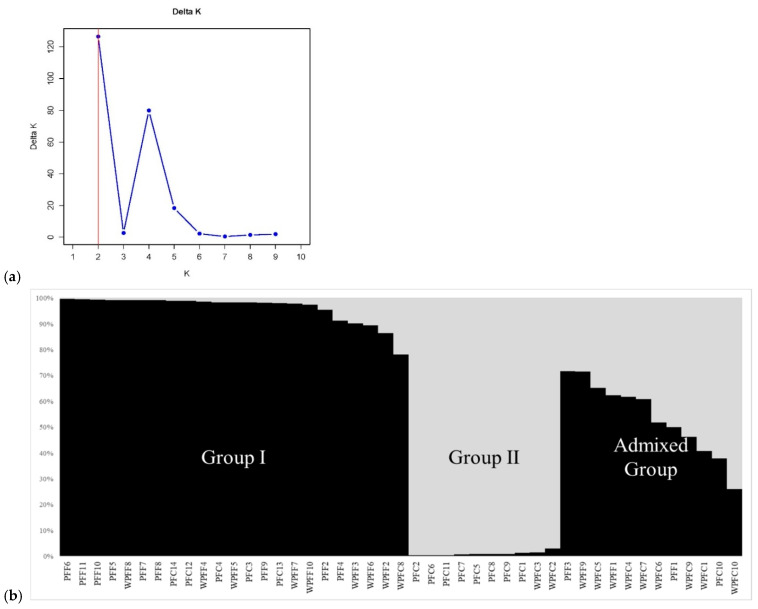
Magnitude of ∆*K* as a function of *K*; the peak value of ∆*K* was at *K* = 2 (**a**). Population structure of four *Perilla* types inferred from 45 accessions using 70 SSR markers at *K* = 2 (**b**).

Population structure analysis (PSA) revealed a pronounced peak at *K* = 2 (Figure 2a). By applying a membership probability threshold of 0.75, the accessions were assigned into two major genetic groups (Groups I and II) and an admixed group (Figure 2b). Group I comprised 23 accessions, including 9 PFF cultivars, 5 PFC cultivars, 8 WPFF accessions, and 1 WPFC accession. Group II consisted of 10 accessions, including 8 PFC cultivars and 2 WPFC accessions. The remaining 12 accessions formed the admixed group, containing 2 PFF cultivars, 1 PFC cultivar, 2 WPFF accessions, and 7 WPFC accessions. This pattern indicates partial genetic differentiation between cultivated and weedy forms, accompanied by substantial admixture, particularly among the WPFC accessions.

**Figure 3:**
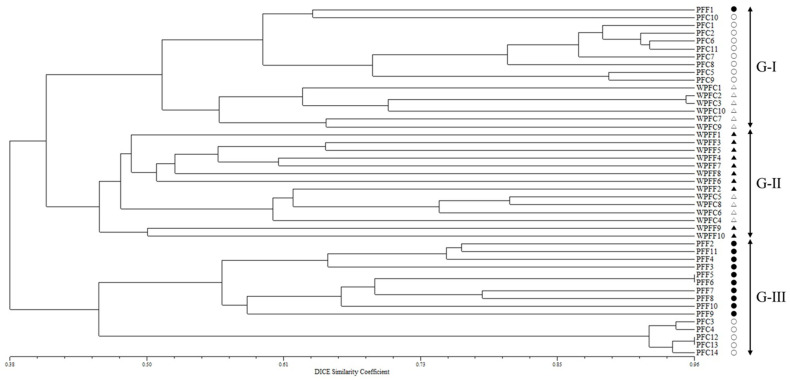
UPGMA dendrogram of 45 *Perilla* accessions (11 PFF, 14 PFC, 10 WPFF, and 10 WPFC) collected from South Korea and Japan, constructed using 70 newly developed *Perilla* SSR markers (●, PFF; ○, PFC; ▲, WPFF; △, WPFC).

Based on the phylogenetic analysis, the 45 accessions were classified into three major clusters at a genetic similarity (GS) threshold of 45% (Figure 3). Group I included 1 PFF cultivar, 9 PFC cultivars, and 6 WPFC accessions; Group II consisted exclusively of weedy accessions (10 WPFF and 4 WPFC); and Group III contained 10 PFF and 5 PFC cultivars. Overall, most of the cultivated PFF and PFC cultivars were clearly separated from the weedy WPFF and WPFC accessions, although the two weedy groups were not distinctly differentiated from each other. In addition, the cultivated PFF and PFC cultivars were generally well separated, with only a few exceptions.

**Figure 4:**
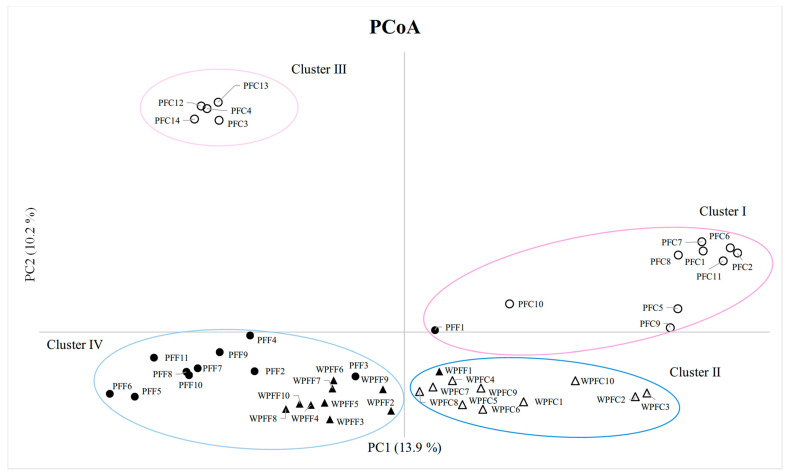
Principal coordinates analysis (PCoA) of 45 *Perilla* accessions (11 PFF, 14 PFC, 10 WPFF, and 10 WPFC) collected from South Korea and Japan based on 70 newly developed *Perilla* SSR markers (●, PFF; ○, PFC; ▲, WPFF; △, WPFC).

To further resolve the genetic differentiation among the four *Perilla* types (PFF, PFC, WPFF, and WPFC), principal coordinate analysis (PCoA) was conducted based on genetic distance. The first two principal coordinates explained 24.1% of the total genetic variation, with PC1 and PC2 accounting for 13.9% and 10.2%, respectively (Figure 4). The PCoA scatter plot revealed four major clusters. Cluster I included 9 PFC cultivars (PFC1, PFC2, PFC5–PFC11) together with 1 PFF cultivar (PFF1), while Cluster II consisted of 1 WPFF accession (WPFF1) and all 10 WPFC accessions (WPFC1–WPFC10). Cluster III comprised 5 PFC cultivars (PFC3, PFC4, PFC12, PFC13, and PFC14), whereas Cluster IV contained 10 PFF cultivars (PFF2–PFF11) and 9 WPFF accessions (WPFF2–WPFF10). In general, the PFC and WPFC accessions were distributed on the positive side of PC1, whereas the PFF and WPFF accessions were located on the negative side. However, several accessions (PFF1, WPFF1, PFC3, PFC4, PFC12, PFC13, and PFC14) showed intermediate positions and were not clearly separated. Overall, the clustering patterns observed in the PCoA were largely congruent with those revealed by the UPGMA dendrogram (Figure 3).

The analysis of molecular variance (AMOVA) assessing genetic differentiation among and within the 45 *Perilla* accessions representing the four *Perilla* types (PFF, PFC, WPFF, and WPFC) is presented in Supplementary Figure S2. The results indicated that 28% of the total genetic variation was attributable to differences among the four *Perilla* types, whereas the remaining 72% was explained by variation within the types.

### 2.4. Marker–Trait Associations for Ten Morphological Traits in 45 Perilla Accessions Using Q GLM and Q + K MLM

Marker–trait associations detected as significant in both the Q GLM and Q + K MLM models were regarded as robust associations. Association mapping analyses (AMA) were performed between 70 SSR markers and ten morphological traits (five qualitative and five quantitative traits) across the 45 *Perilla* accessions using both the Q-based general linear model (Q GLM) and the Q + K mixed linear model (Q + K MLM). Using the Q GLM, a total of 226 significant marker–trait associations involving 59 SSR markers were detected across the ten traits (Supplementary Table S3). At significance thresholds of *p* < 0.05 or *p* < 0.01, the number of associated markers per trait was 7 for QL1, 10 for QL2, 18 for QL3, 25 for QL4, 28 for QL5, 30 for QN1, 18 for QN2, 10 for QN3, 39 for QN4, and 41 for QN5.

To reduce false-positive associations arising from population structure and kinship, AMA was further conducted using the more stringent Q + K MLM. Compared to the Q GLM, the Q + K MLM identified substantially fewer significant associations. Using this model, 26 significant marker–trait associations involving 18 SSR markers were detected for the ten morphological traits at *p* < 0.05 or *p* < 0.01 (Supplementary Table S3). The number of associated markers per trait under the Q + K MLM was two for QL1, one for QL2, three for QL3, four for QL4, one for QL5, one for QN1, two for QN2, three for QN3, three for QN4, and six for QN5. The reduced number of significant associations detected by the MLM model likely reflects its ability to more effectively control false positives by accounting for both population structure and kinship relationships among accessions.

Comparison of the results obtained from the two models identified 23 overlapping significant marker–trait associations (SMTAs) involving 16 SSR markers (Table 3). Since these SMTAs were consistently detected by both approaches, they are considered robust associations. Among these SMTAs, two markers were associated with QL1; one with QL2; three with QL3; two with QL4; one with QL5; one with QN1; two with QN2; three with QN3; two with QN4; and six with QN5. Notably, several SSR markers were associated with multiple qualitative or quantitative traits: KNUPF192 with QL1 and QN2; KNUPF230 with QL3, QN4, and QN5; KNUPF207 with QN4 and QN5; KNUPF238 with QL4, QN4, and QN5; and KNUPF202 with QN2 and QN3. This suggests potential pleiotropic effects or tight genetic linkage among loci for related phenotypes.

## 3. Discussion

### 3.1. Genetic Variation and Morphological Differentiation Between Cultivated and Weedy Types of the Two Perilla Varieties

Morphological variation between the cultivated and weedy types of the two *Perilla* varieties provides important taxonomic evidence for understanding their evolutionary history and domestication processes [2,16]. In East Asia, *Perilla* has traditionally been classified into two cultivated varieties, PFF and PFC, based on their primary uses as seed oil crops or as leafy vegetables and medicinal plants, respectively [2,16,29]. Although true wild relatives of cultivated *Perilla* have not been identified in this region, corresponding weedy forms (WPFF and WPFC) were reported for both varieties by Nitta and Ohnishi (1999) [30] and Lee and Ohnishi (2001) [16]. In South Korea, weedy *Perilla* accessions are commonly observed in disturbed habitats such as field margins, farmhouse surroundings, and roadside environments, where they are often regarded as descendants of escaped or hybridized cultivated forms. In contrast, clearly identifiable weedy populations have been reported less frequently in Japan. Previous studies have suggested that the domestication status of the two *Perilla* varieties differs substantially. For example, Lee and Ohnishi (2001, 2003) [16,31] reported little morphological or AFLP differentiation between Japanese PFC cultivars and weedy WPFC accessions from South Korea, suggesting that PFC may represent a relatively weakly differentiated or semi-domesticated form.

In the present study, a comprehensive morphological trait analysis was therefore conducted as a preliminary step toward developing molecular markers associated with phenotypic traits distinguishing the cultivated and weedy forms of the two *Perilla* varieties. Among the 20 qualitative and quantitative traits examined, five qualitative (QL1–QL5) and five quantitative (QN1–QN5) traits were identified as the most effective in discriminating between the cultivated and weedy forms [28]. The cultivated *Perilla* accessions (PFF and PFC) exhibited greater phenotypic diversity in qualitative traits, such as leaf pigmentation and seed hardness, reflecting directional selection under human cultivation, whereas the weedy accessions (WPFF and WPFC) showed more uniform traits consistent with stabilizing selection in unmanaged environments. Seed-related traits were particularly informative indicators of domestication. Cultivated PFF was characterized by soft seeds, larger seed size, and higher 100-seed weight, whereas cultivated PFC and the weedy accessions (WPFF and WPFC) consistently exhibited hard seeds and markedly lower seed mass (Table 1). These patterns likely reflect divergent selection pressures, with artificial selection enhancing seed-related traits in cultivated PFF, whereas the reduced seed mass observed in cultivated PFC and the weedy accessions (WPFF and WPFC) suggests weak or absent directional selection in non-domesticated populations. Overall, these morphological differences are consistent with domestication signatures reported for seed-bearing crops [10,32].

Genetic diversity analysis using 70 SSR loci detected a total of 330 alleles among the 45 *Perilla* accessions, with 2–12 alleles per locus (mean = 4.7). Approximately 25% of the alleles were private alleles unique to specific groups, while rare and intermediate-frequency alleles accounted for 34% and 27%, respectively, indicating substantial allelic variation across the *Perilla* accessions (Supplementary Figure S1). Mean GD values for the PFF, WPFF, PFC, and WPFC accessions were 0.375, 0.489, 0.383, and 0.406, respectively, with corresponding PIC values of 0.322, 0.437, 0.323, and 0.356 (Supplementary Table S2). The KNUPF194 marker exhibited fewer allelic fragments in cultivated PFF accessions than in cultivated PFC and weedy WPFF and WPFC accessions (Supplementary Figure S3). This allelic pattern clearly distinguished most cultivated PFF accessions from cultivated PFC and weedy accessions, although several exceptions were observed. Despite the relatively limited number of accessions analyzed, the diversity patterns distinguishing the cultivated and weedy types of the two *Perilla* varieties were consistent with those reported in previous studies using AFLP [1,31] and SSR markers [24,26,33].

As previously reported by Lee and Ohnishi (2003) [31], the higher genetic variation observed in weedy accessions likely reflects weak artificial selection and the retention of ancestral variation, whereas the reduced diversity in cultivated accessions may result from domestication-related bottlenecks and directional selection. Similar patterns have been reported in many domesticated crops, in which cultivated populations typically exhibit lower genetic diversity than their wild or weedy counterparts [31,34,35,36,37]. However, an important limitation of the present study is the geographical imbalance of the sampled accessions. Most cultivated PFC cultivars analyzed in this study were obtained from Japan, whereas the weedy accessions were collected from South Korea. Consequently, varietal identity, domestication status, and geographic origin are partially confounded in the current sampling design, which may influence the interpretation of the population structure and clustering analyses. For example, the clustering pattern observed at *K* = 2 in the STRUCTURE analysis may indicate differentiation between cultivated and weedy types, but it may also partly reflect geographic genetic differentiation between Korean and Japanese populations. Similar patterns were observed in the PCoA and UPGMA analyses, where the separation between cultivated and weedy accessions may likewise be influenced by geographic origin. Furthermore, some of the marker–trait associations detected in the association mapping analysis may partly reflect underlying population structure rather than direct causal relationships with domestication-related traits.

Therefore, the genetic differentiation and marker–trait associations identified in this study should be interpreted with caution. Future studies incorporating more geographically balanced sampling, including additional cultivated and weedy accessions from both South Korea and Japan, will be necessary to more clearly disentangle the relative contributions of domestication, varietal differentiation, and geographic population structure.

### 3.2. Trait Correlations, Population Structure, and Phylogenetic Relationships of Cultivated and Weedy Types of Two Perilla Varieties from South Korea and Japan

Correlation analysis revealed strong relationships among several phenotypic traits, particularly among seed-related characteristics such as seed color, seed hardness, seed size, and seed weight. Notably, seed size (QN4) and 100-seed weight (QN5) showed an exceptionally high correlation (r = 0.932), indicating close functional and developmental integration. Such correlations likely reflect coordinated selection on seed traits during the domestication process, particularly in PFF, which has been domesticated primarily as an oil crop. In contrast, cultivated PFC and the weedy accessions tend to produce relatively smaller seeds while maintaining higher reproductive output, suggesting different resource allocation strategies between seed-type and leaf-type cultivars. Trade-offs between reproductive output and individual seed investment have been widely reported in plant life-history studies [38,39,40]. Consistent with this pattern, cultivated PFF exhibits increased seed size as a result of artificial selection, whereas cultivated PFC and the weedy accessions maintain higher reproductive output with relatively smaller seeds [16,31]. Because cultivated PFC is primarily grown for leaf production in Japan, selection pressure on seed-related traits may have been relatively weak. Notably, the PFC cultivars displayed pronounced genetic heterogeneity, with several accessions (e.g., PFC3, PFC4, and PFC12–14) forming distinct clusters in both PCoA and UPGMA analyses, whereas others clustered with additional PFC and PFF cultivars. Collectively, these patterns suggest that *Perilla* domestication involved shifts in resource allocation strategies, favoring increased investment in individual seeds over reproductive fecundity in seed-type cultivars. Furthermore, the observed associations between qualitative morphological traits (e.g., leaf and flower color) and quantitative seed characteristics suggest that easily observable phenotypes may serve as proxies for underlying life-history strategies, thereby enhancing the efficiency of phenotypic selection and germplasm evaluation in breeding programs.

On the other hand, PSA consistently resolved two major genetic groups corresponding to the two *Perilla* varieties, reflecting their deep evolutionary divergence (Figure 2). Despite this clear varietal separation, extensive admixture was observed between the cultivated and weedy accessions within each variety. The absence of distinct genetic boundaries between the cultivated and weedy forms suggests ongoing or historical gene flow, incomplete reproductive isolation, and repeated feralization events [32]. This interpretation is further supported by the AMOVA results (Supplementary Figure S2), which showed that genetic variation among the 45 *Perilla* accessions was predominantly structured within rather than among the four *Perilla* types (PFF, PFC, WPFF, and WPFC). Only 28% of the total genetic variation was attributable to differences among the *Perilla* types, whereas the majority (72%) resided within the types, indicating substantial intra-type genetic diversity. Consistent with these findings, UPGMA clustering and PCoA analyses (Figure 3 and Figure 4) revealed broadly overlapping yet partially differentiated genetic clusters associated with varietal identity and domestication status. In both analyses, the cultivated PFF and PFC cultivars generally formed distinct groups relative to the weedy WPFF and WPFC accessions and to each other. However, several exceptions were observed, and genetic differentiation between the two weedy groups remained weak. Notably, the close clustering of several weedy accessions with their cultivated counterparts suggests that some weedy forms may have arisen repeatedly through natural hybridization between the cultivated and weedy populations or through derivation from wild progenitors, as previously reported by Lee and Ohnishi (2003) [31], Sa et al. (2013) [41], and Fu et al. (2023) [42]. Such genetic patterns are characteristic of crop–weed complexes, in which taxonomic and genetic boundaries are often diffuse due to bidirectional gene flow and recurrent escape from cultivation, as documented in brassicas, rice, and other domesticated crops [43,44]. Taken together, the genetic patterns observed in this study suggest that differentiation in *Perilla* likely occurred along a crop–weed continuum characterized by recurrent gene flow and incomplete divergence between cultivated and weedy populations.

### 3.3. Marker–Trait Associations and Genetic Architecture of Agronomic Traits

In this study, AMA was conducted using genotypic data from 70 newly developed *Perilla* SSR markers and phenotypic data for ten agronomic traits across 45 accessions representing cultivated and weedy types of two *Perilla* varieties. AMA performed using both the Q GLM and the Q + K MLM models identified a subset of marker–trait associations that were consistently detected across both models (Supplementary Table S3). As observed in previous association-mapping studies, the number of significant associations was substantially reduced under the more stringent Q + K MLM model, demonstrating the importance of accounting for population structure and kinship to minimize false-positive associations [45]. Across both models, a total of 23 overlapping SMTAs involving 16 SSR markers were identified (Table 3). Associations consistently detected across statistical models are generally considered to represent loci with robust effects across diverse genetic backgrounds. The moderate coefficients of determination (*R*^2^) observed for most SMTAs suggest that the evaluated traits are governed by a polygenic architecture comprising multiple loci with small to moderate effects, rather than by single major-effect genes [46]. Notably, several SSR markers were significantly associated with multiple qualitative or quantitative traits, indicating potential pleiotropic effects or tight genetic linkage among the loci for related phenotypes. Specifically, KNUPF192 was associated with QL1 and QN2; KNUPF202 with QN2 and QN3; KNUPF207 with QN4 and QN5; KNUPF230 with QL3, QN4, and QN5; and KNUPF238 with QL4, QN4, and QN5. Several pairs of marker-associated traits, namely QN2 and QN3, QN4 and QN5, and QL4 and QN5, exhibited strong phenotypic correlations (Figure 1; Supplementary Table S1), suggesting coordinated genetic regulation. However, it should be noted that the association analysis in this study was conducted using a relatively limited number of accessions. Small population sizes may reduce statistical power and increase the likelihood of detecting spurious marker–trait associations [47]. Because of this limitation, strict multiple testing correction methods, such as the false discovery rate (FDR), were not applied, and the identified associations should therefore be interpreted with caution. Accordingly, the SMTAs identified in this study should be regarded as preliminary signals rather than definitive causal relationships. Further validation using larger populations, independent germplasm panels, or integrative approaches combining high-density genomic markers, genome-wide association analyses, and functional genomic studies will be necessary to elucidate the genetic basis of these traits.

Previous studies have reported *Perilla* SSR primer sets associated with leaf- and seed-related traits in cultivated and weedy accessions of two *Perilla* varieties [10,17,25]. Building on this foundation, the present study expands the available genomic resources by applying 70 newly developed SSR primer sets [48] to the integrated analyses of genetic diversity, population structure, and association mapping. Despite these advances, informative SSR markers for *Perilla* remain relatively limited, constraining detailed evaluations of GD, PIC, PSA, and AMA across the cultivated and weedy accessions. Accordingly, the genetic information generated in this study represents a valuable resource for future genetic and breeding research for *Perilla*. In particular, five SSR markers (KNUPF192, KNUPF202, KNUPF207, KNUPF230, and KNUPF238) associated with multiple qualitative and quantitative traits were identified as candidate markers requiring further validation for potential phenotypic improvement in both cultivated and weedy *Perilla* types. Markers linked to seed-related traits (QN4 and QN5) may be particularly relevant for improving PFF cultivars with enhanced oil yield, whereas markers associated with qualitative leaf traits (QL1 and QL2) may contribute to the development of improved PFC cultivars with desirable leaf characteristics and potentially enhanced bioactive compound content.

Collectively, these findings provide an integrated view of phenotypic variation, genetic diversity, and trait architecture across cultivated and weedy types of the two *Perilla* varieties. The combined morphological, population genetic, and association analyses highlight the dynamic evolutionary interplay between domesticated and weedy *Perilla* types. Nevertheless, several limitations should be acknowledged. First, the total number of accessions analyzed (n = 45) is relatively small for association mapping analyses. Such a limited sample size may reduce the statistical power to detect true marker–trait associations and increase the likelihood of false-positive signals. In addition, correlations among morphological traits may be overestimated when the number of observations is limited. Therefore, the marker–trait associations identified in this study should be regarded as preliminary and interpreted with caution. Second, the geographical origins of the sampled germplasm were not fully balanced. Most cultivated PFC accessions were obtained from Japan, whereas the weedy accessions were collected from South Korea. As a result, geographical origin, varietal identity, and domestication status are partially confounded in the present dataset. Consequently, some of the genetic differentiation patterns and marker–trait associations observed in this study may reflect geographic population structure rather than domestication-related divergence alone. Future studies incorporating larger germplasm collections with more balanced geographical representation will be necessary to disentangle the relative contributions of domestication, varietal differentiation, and geographic structure. In addition, the candidate marker–trait associations identified in this study should be validated using larger populations and independent germplasm panels.

## 4. Materials and Methods

### 4.1. Plant Materials and Morphological Characteristics of Cultivated and Weedy Perilla Accessions

A total of 45 *Perilla* accessions representing cultivated and weedy *Perilla* forms were analyzed. These accessions comprised 11 *P. frutescens* var. *frutescens* (PFF), 14 *P. frutescens* var. *crispa* (PFC), 10 weedy PFF (WPFF), and 10 weedy PFC (WPFC) accessions collected from South Korea and Japan (Supplementary Table S4). Among the cultivated accessions, 10 PFF cultivars were developed by the National Institute of Crop and Food Science, Rural Development Administration (RDA), South Korea, whereas 1 PFF cultivar and all of the PFC cultivars were developed by a private seed company in Japan. The weedy accessions (WPFF and WPFC) were collected in South Korea by the research team, as previously described by Lee et al. (2026) [28].

Morphological data for the 45 *Perilla* accessions were obtained from Lee et al. (2026) [28]. The dataset included five qualitative traits (adaxial leaf color (QL1), abaxial leaf color (QL2), flower color (QL3), seed color (QL4), and seed hardness (QL5)), and five quantitative traits (plant height (QN1), length of inflorescence (QN2), number of florets (QN3), seed size (QN4), and 100-seed weight (QN5) (Table 1). These traits were previously demonstrated to effectively discriminate between cultivated and weedy accessions based on principal component analysis and were therefore selected for subsequent trait association analyses in the present study.

### 4.2. DNA Extraction and SSR Analysis

Genomic DNA extracted from young leaf tissue was used as a template for PCR amplification using *Perilla*-specific SSR primer sets. A total of 70 SSR primer sets, developed previously by Lee et al. (2025) [48], were employed in this study (Supplementary Table S5). PCR amplifications were performed using an Ex Taq PCR kit (Takara, Ohtsu, Japan) in a final reaction volume of 20 µL containing 20 ng of genomic DNA, 1× PCR buffer, 0.5 µM of each forward and reverse primer, 0.2 mM dNTPs, and 1 U of Taq DNA polymerase (Biotools, Madrid, Spain). Thermal cycling conditions consisted of an initial denaturation at 94 °C for 5 min, followed by 35 cycles of denaturation at 94 °C for 30 s, annealing at 55 °C for 30 s, extension at 72 °C for 1 min 30 s, and a final extension at 72 °C for 5 min. The amplified PCR products were initially separated using a mini vertical electrophoresis system (MGV-202–33, CBS Scientific Company, San Diego, CA, USA). For fragment analysis, 3 µL of each PCR product was mixed with 3 µL of loading buffer containing 98% formamide, 0.02% xylene cyanol, 0.02% bromophenol blue, and 5 mM NaOH. Following denaturation and rapid cooling, 2 µL of the mixture was loaded onto a 6% denaturing polyacrylamide gel (7.5 M urea; 19:1 acrylamide:bisacrylamide) and electrophoresed in 0.5× TBE buffer at 250 V for 30 min. The DNA fragments were visualized by ethidium bromide staining, as shown in Supplementary Figure S3.

### 4.3. Data Analysis

The morphological trait data were analyzed using MetaboAnalyst 6.0 to generate correlation matrices among the traits. The SSR alleles amplified by the 70 *Perilla* SSR primer sets across the 45 cultivated and weedy accessions were scored as present (1) or absent (0). Genetic variation was evaluated based on the number of alleles, major allele frequency (MAF), genetic diversity (GD), and polymorphism information content (PIC). Genetic diversity (GD) within each group of *Perilla* accessions was estimated using Nei’s genetic diversity index [49], calculated as GD = 1 − ∑ *P_i_*^2^, where *P_i_* represents the frequency of the *i*th SSR allele within a group *of Perilla accessions*. For all 45 *Perilla* accessions and 70 *Perilla* SSR loci, the number of alleles, major allele frequency (MAF), and polymorphism information content (PIC) were calculated using PowerMarker version 3.25 [49,50]. Genetic similarity (GS) between pairs of cultivated and weedy accessions was estimated using the Dice similarity coefficient [51]. The resulting similarity matrix was used to construct a dendrogram using the unweighted pair group method with arithmetic mean (UPGMA) and to perform principal coordinate analysis (PCoA) based on the SAHN clustering algorithm implemented in NTSYS-pc version 2.1 [52].

To further examine the genetic relationships among the four *Perilla* types (PFF, PFC, WPFF, and WPFC), a phylogenetic tree was constructed using POPGENE version 1.32 [53] based on Nei’s genetic distance [54] and visualized using MEGA-X version 11.0.11 [55]. Population structure and phylogenetic relationships among the cultivated and weedy accessions of the two *Perilla* varieties from South Korea and Japan were investigated using 45 accessions representing the four *Perilla* types (PFF, PFC, WPFF, and WPFC). The population structure of the 45 *Perilla* accessions was inferred using a Bayesian clustering approach implemented in STRUCTURE version 2.3 [56]. The optimal number of genetic clusters (*K*) was determined using the *ΔK* method of Evanno et al. (2005) [57] implemented in the web-based program STRUCTURE HARVESTER. Analysis of molecular variance (AMOVA) was conducted using GenAlEx version 6.5 [58]. Finally, association mapping analysis (AMA) was performed using TASSEL version 3.0 [59] to identify marker–trait associations for the five qualitative and five quantitative morphological traits. Both a general linear model incorporating population structure (Q GLM) and a mixed linear model incorporating population structure and kinship (Q + K MLM) were applied following Mathiang et al. (2023) [45]. Because the number of accessions analyzed in this study was relatively limited, a strict multiple testing correction, such as the false discovery rate (FDR), was not applied. Therefore, the detected marker–trait associations should be interpreted cautiously and regarded as preliminary results requiring further validation. Basic statistical analyses were conducted using Microsoft Excel 2016, and correlation analyses of agronomic traits were performed using MetaboAnalyst 6.0 (Montreal, QC, Canada, https://www.metaboanalyst.ca (accessed on 26 January 2026)). The analysis showed that numerous trait pairs were significantly correlated, displaying either positive or negative relationships at the 0.05 and 0.01 significance levels.

## 5. Conclusions

*Perilla frutescens* comprises two varieties that are distinguished by their morphological characteristics and patterns of utilization in East Asia. In this study, 70 polymorphic SSR markers generated 330 alleles across 45 *Perilla* accessions representing cultivated (PFF and PFC) and weedy (WPFF and WPFC) forms from South Korea and Japan. The weedy accessions consistently exhibited higher genetic diversity than the cultivated forms, indicating that weedy populations retain greater levels of ancestral genetic variation. Phylogenetic analysis separated the 45 accessions into three major clusters, largely distinguishing cultivated and weedy types. However, several weedy accessions clustered together with cultivated groups, suggesting that some weedy forms may have arisen repeatedly through natural hybridization between cultivated and weedy populations. Association mapping using Q GLM and Q + K MLM models identified 23 significant marker–trait associations across 16 SSR markers. Five markers (KNUPF192, KNUPF202, KNUPF207, KNUPF230, and KNUPF238) were associated with multiple qualitative and quantitative traits, particularly seed size/weight and leaf pigmentation. These loci represent promising candidate markers for future marker-assisted selection aimed at improving seed yield traits in PFF cultivars and desirable leaf characteristics in PFC cultivars. Nevertheless, the relatively small number of accessions analyzed in this study may limit the statistical power of the association analysis. Therefore, the detected marker–trait associations should be considered preliminary and require validation using larger germplasm collections, independent populations, or genome-wide marker systems. Future studies incorporating *Perilla* accessions with more balanced geographical origins will be necessary to better disentangle the effects of domestication, varietal differentiation, and geographic population structure. Overall, this study provides an integrated understanding of the genetic diversity, population structure, and trait-associated loci across the cultivated and weedy forms of the two *Perilla* varieties.

## Figures and Tables

**Table 1:** Summary of morphological characteristics for five qualitative and five quantitative traits in 45 *Perilla* accessions representing four *Perilla* types (PFF, PFC, WPFF, and WPFC).

Morphological Character	PFF (n = 11)	WPFF (n = 10)	PFC (n = 14)	WPFC (n = 10)
QL1 Leaf adaxial color	Light green (3), green (7), greenish red (1)	Light green (3), green (4), deep green (3)	Light green (5), green (8), purple (1)	Green (5), greenish red (2), purple (3)
QL2 Leaf abaxial color	Light green (4), green (3), greenish red (2), purple (2)	Light green (3), green (4), deep green (1), greenish red (1), purple (1)	Light green (2), green (4), greenish red (6), purple (2)	Purple (10)
QL3 Flower color	White (10), light pink (1)	White (7), light pink (2), pink (1)	White (11), pink (3)	Light pink (1), pink (9)
QL4 Seed color	White (3), gray (2), light brown (2), brown (4)	Brown (4), dark brown (6)	Gray (2), light brown (3), brown (6), dark brown (3)	Light brown (2), brown (6), dark brown (2)
QL5 Seed hardness	Hard (5), soft (6)	Hard (10)	Hard (14)	Hard (10)
QN1 Plant height (cm)	140.0 (113.8~166.8)	141.3 (130.3~154.3)	129.5 (104.5~143.7)	117.1 (97.7~128.5)
QN2 Length of inflorescence (cm)	7.1 (5.4~9.5)	10.6 (7.3~15.9)	10.3 (7.4~18.3)	11.9 (7.6~20.0)
QN3 Number of florets (number)	34.7 (24.0~46.7)	40.5 (25.4~54.7)	36.5 (26.7~64.0)	45.1 (36.0~68.0)
QN4 Seed size (cm)	1.95 (1.38~2.34)	1.13 (0.87~1.45)	1.20 (0.95~1.58)	0.97 (0.85~1.31)
QN5 100-seed weight (g)	0.271 (0.184~0.414)	0.108 (0.070~0.152)	0.115 (0.074~0.158)	0.082 (0.070~0.129)

Data for five qualitative and five quantitative traits previously published by Lee et al. (2026) [28]. For quantitative traits, values indicate the mean, minimum, and maximum measured within each type.

**Table 2:** Estimates of allele number, MAF, GD, and PIC for 70 SSR primer sets in 45 *Perilla* accessions (11 PFF, 14 PFC, 10 WPFF, and 10 WPFC) collected from South Korea and Japan.

Marker	No. Allele	MAF	GD	PIC	Marker	No. Allele	MAF	GD	PIC
KNUPF169	4	0.533	0.596	0.525	KNUPF206	2	0.533	0.498	0.374
KNUPF170	6	0.489	0.679	0.635	KNUPF207	6	0.711	0.471	0.447
KNUPF171	4	0.400	0.657	0.587	KNUPF208	3	0.622	0.486	0.388
KNUPF172	5	0.644	0.511	0.449	KNUPF209	3	0.800	0.328	0.287
KNUPF173	4	0.600	0.568	0.513	KNUPF210	3	0.511	0.520	0.406
KNUPF174	3	0.533	0.518	0.405	KNUPF211	3	0.867	0.236	0.217
KNUPF175	4	0.622	0.514	0.436	KNUPF212	4	0.622	0.501	0.414
KNUPF176	6	0.467	0.632	0.565	KNUPF213	3	0.867	0.236	0.217
KNUPF177	7	0.311	0.773	0.738	KNUPF214	2	0.756	0.369	0.301
KNUPF178	6	0.489	0.680	0.638	KNUPF215	5	0.311	0.759	0.717
KNUPF179	8	0.600	0.610	0.588	KNUPF216	3	0.733	0.411	0.355
KNUPF180	11	0.244	0.834	0.814	KNUPF217	3	0.489	0.600	0.519
KNUPF181	4	0.489	0.595	0.513	KNUPF218	6	0.356	0.735	0.691
KNUPF182	4	0.556	0.560	0.478	KNUPF219	7	0.511	0.687	0.659
KNUPF183	9	0.378	0.776	0.749	KNUPF220	5	0.556	0.595	0.533
KNUPF184	6	0.289	0.765	0.726	KNUPF221	4	0.422	0.665	0.597
KNUPF185	5	0.400	0.703	0.650	KNUPF222	5	0.356	0.751	0.709
KNUPF186	9	0.289	0.778	0.745	KNUPF223	2	0.600	0.480	0.365
KNUPF187	6	0.311	0.740	0.693	KNUPF224	4	0.800	0.335	0.303
KNUPF188	7	0.356	0.767	0.734	KNUPF225	3	0.711	0.423	0.350
KNUPF189	10	0.267	0.809	0.783	KNUPF226	2	0.911	0.162	0.149
KNUPF190	11	0.289	0.833	0.814	KNUPF227	5	0.622	0.556	0.510
KNUPF191	12	0.267	0.845	0.829	KNUPF228	2	0.756	0.369	0.301
KNUPF192	4	0.533	0.615	0.555	KNUPF229	3	0.578	0.575	0.510
KNUPF193	5	0.467	0.615	0.540	KNUPF230	3	0.533	0.551	0.456
KNUPF194	4	0.467	0.631	0.559	KNUPF231	3	0.533	0.518	0.405
KNUPF195	2	0.533	0.498	0.374	KNUPF232	5	0.311	0.719	0.666
KNUPF196	4	0.600	0.526	0.444	KNUPF233	2	0.756	0.369	0.301
KNUPF197	5	0.622	0.526	0.459	KNUPF234	2	0.711	0.411	0.326
KNUPF198	4	0.556	0.546	0.456	KNUPF235	8	0.422	0.698	0.649
KNUPF199	4	0.511	0.558	0.463	KNUPF236	3	0.600	0.512	0.419
KNUPF200	3	0.733	0.402	0.337	KNUPF237	4	0.378	0.677	0.609
KNUPF201	7	0.533	0.660	0.626	KNUPF238	6	0.600	0.588	0.549
KNUPF202	4	0.467	0.631	0.559					
KNUPF203	4	0.511	0.558	0.463	**Mean**	4.7	0.543	0.566	0.506
KNUPF204	2	0.956	0.085	0.081	**Max**	12	0.956	0.845	0.829
KNUPF205	3	0.889	0.203	0.193	**Min**	2	0.244	0.085	0.081

**Table 3:** Significant marker–trait associations (SMTAs) for 10 traits identified using the GLM and MLM models in 45 *Perilla* accessions representing four *Perilla* types (PFF, PFC, WPFF, and WPFC).

Trait	Marker	GLM	MLM
QL1 Leaf adaxial color	KNUPF192	**	*
	KNUPF216	**	*
QL2 Leaf abaxial color	KNUPF205	**	**
QL3 Flower color	KNUPF224	*	*
	KNUPF230	**	*
	KNUPF236	**	**
QL4 Seed color	KNUPF207	**	*
	KNUPF238	**	*
QL5 Seed hardness	KNUPF195	*	*
QN1 Plant height	KNUPF169	**	*
QN2 Length of inflorescence	KNUPF192	*	*
	KNUPF202	**	**
QN3 Number of florets	KNUPF179	*	*
	KNUPF183	**	*
	KNUPF202	**	*
QN4 Seed size	KNUPF230	**	*
	KNUPF238	**	*
QN5 100-seed weight	KNUPF190	**	*
	KNUPF207	**	*
	KNUPF209	*	*
	KNUPF230	**	*
	KNUPF233	**	*
	KNUPF238	**	**

* *p* < 0.05; ** *p* < 0.01.

## Data Availability

The original contributions presented in this study are included in the article/Supplementary Materials. Further inquiries can be directed to the corresponding author.

## Supplementary Materials

The following supporting information can be downloaded at https://www.mdpi.com/article/10.3390/plants15081273/s1. Supplementary Figure S1. Histogram of allele frequency for a total of 330 alleles in the 45 *Perilla* accessions collected from South Korea and Japan. Supplementary Figure S2. Percentages of molecular variance based on SSR markers among the 45 *Perilla* accessions representing cultivated and weedy types collected from South Korea and Japan. Supplementary Figure S3. Representative SSR profiles of the four *Perilla* types (PFF, PFC, WPFF, and WPFC) collected from South Korea and Japan. The profiles were resolved on a 6% native polyacrylamide gel using the SSR primer KNUPF194. Supplementary Table S1. Pearson correlation coefficients among the 10 morphological traits measured in the 45 *Perilla* accessions. Supplementary Table S2. Genetic variation detected by each SSR primer among four groups (PFF, PFC, WPFF, and WPFC) within 45 *Perilla* accessions collected from South Korea and Japan. Supplementary Table S3. Information on marker–trait associations (MTAs) identified using the GLM and MLM approaches in the 45 *Perilla* accessions representing four *Perilla* types (PFF, PFC, WPFF, and WPFC). Supplementary Table S4. Summary of the 45 accessions (11 PFF, 14 PFC, 10 WPFF, and 10 WPFC) representing cultivated and weedy types of the two *Perilla* varieties collected from South Korea and Japan. Supplementary Table S5. The 70 SSR primer sets used for genetic diversity analysis in the 45 *Perilla* accessions from South Korea and Japan.
