# Supplementary material for: Genetic Diversity and Marker–Trait Associations in Commercial Cultivars and Weedy Perilla frutescens from South Korea and Japan Based on Morphological Traits and SSR Markers
Source: Plants (Basel). 2026 Apr 21;15(8):1273. doi: 10.3390/plants15081273 (PMC13120271; doi:10.3390/plants15081273)
Supplement: Supplementary file 1 [file plants-15-01273-s001.zip › Supplementary Fig. S1.pdf]

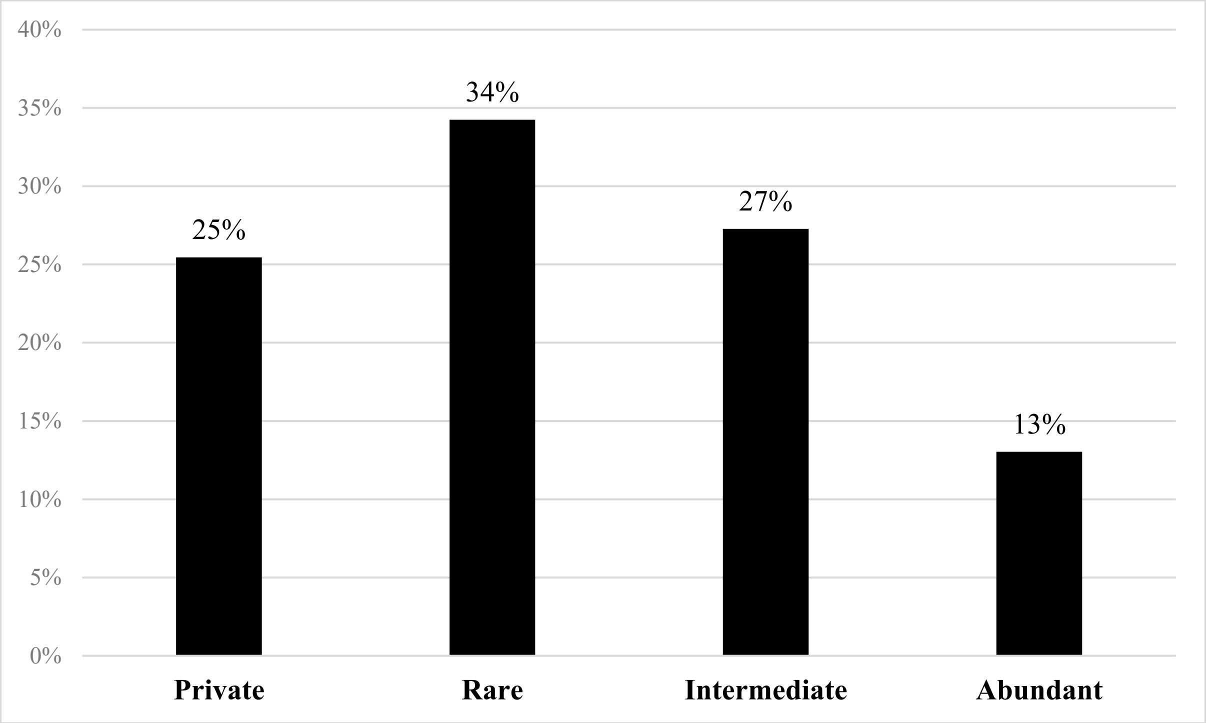

Supplementary Fig. S1. Histogram of allele frequency for a total of 330 alleles in the 45 *Perilla* accessions collected from South Korea and Japan.
