# Supplementary material for: Genetic Diversity and Marker–Trait Associations in Commercial Cultivars and Weedy Perilla frutescens from South Korea and Japan Based on Morphological Traits and SSR Markers
Source: Plants (Basel). 2026 Apr 21;15(8):1273. doi: 10.3390/plants15081273 (PMC13120271; doi:10.3390/plants15081273)
Supplement: Supplementary file 1 [file plants-15-01273-s001.zip › Supplementary Fig. S3.pdf]

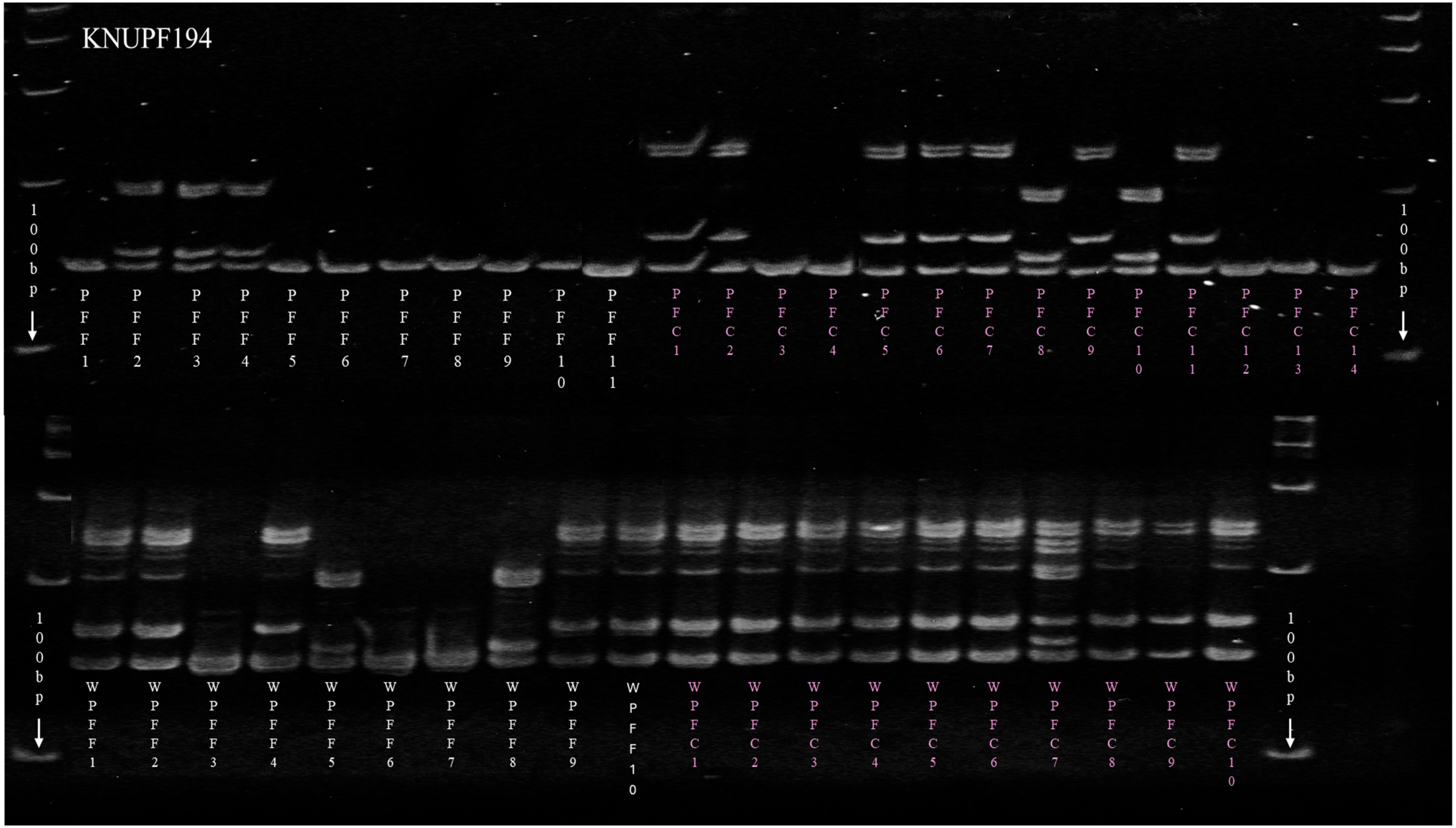

Supplementary Fig. S3. Representative SSR profiles of the four *Perilla* types (PFF, PFC, WPFF, and WPFC) collected from South Korea and Japan. The profiles were resolved on a 6% native polyacrylamide gel using the SSR primer KNUPF194.
